# A novel heterozygous splicing variant in the *EIF4ENIF1* gene in a family with premature ovarian insufficiency and early menopause

**DOI:** 10.3389/fcell.2026.1860224

**Published:** 2026-07-22

**Authors:** Jiaqi Sun, Bo Liu, Mengjiao Hu, Qi Qi, Wenxin Chen, Jianlin Chen, Erkai Liu, Jun Zeng, Longfei Li, Kun Ye, Hualin Huang

**Affiliations:** 1 Reproductive Medicine Center, Department of Obstetrics and Gynecology, The Second Xiangya Hospital, Central South University, Changsha, Hunan, China; 2 Prenatal Diagnosis Center, Department of Obstetrics and Gynecology, The Second Xiangya Hospital, Central South University, Changsha, Hunan, China; 3 Shenzhen Salus BioMed Inc Ltd., Shenzhen, Guangdong, China; 4 Hunan Province Clinical Research Center for Genetic Birth Defects and Rare Diseases, Changsha, Hunan, China

**Keywords:** EIF4ENIF1, exon skipping, premature ovarian insufficiency, RNA splicing, splicing variant

## Abstract

**Background:**

Female reproductive aging is a continuum characterized by the depletion of ovarian follicles, including Early Menopause (EM) and Premature ovarian insufficiency (POI).POI exhibits a strong genetic predisposition. Eukaryotic Translation Initiation Factor 4E nuclear import factor 1 (EIF4ENIF1), a key regulator of gene expression, plays a crucial role in oocyte maturation and follicular development. Accumulating evidence indicates that functional abnormalities in EIF4ENIF1 are closely associated with the onset and progression of POI. Therefore, identifying pathogenic variants in EIF4ENIF1 is of significant importance for elucidating its pathogenic mechanisms in POI and advancing clinical diagnosis.

**Methods:**

Whole exome sequencing (WES) was performed on the patients with POI, EM or DOR to identify potential pathogenic genes. Variant validation was conducted using Sanger sequencing in patients and their family members. Multiple bioinformatics tools were employed to analyze EIF4ENIF1 transcripts and the three-dimensional structures of encoded proteins. The findings were further validated using reverse transcription polymerase chain reaction (RT-PCR).

**Results:**

Through WES analysis, we identified a novel splice-site variant c.1769–2A>G in EIF4ENIF1 gene in the proband. Sanger sequencing confirmed that the proband’s mother, maternal grandmother, and sister also carry this variant. RT-PCR and Sanger sequencing detected exon 11 skipping caused by this splicing variant (p.Ile589fs*18). Protein structure prediction indicated disruption of the C-terminal domain of EIF4ENIF1 protein, leading to significant structural alterations in the truncated protein.

**Conclusion:**

This study highlights the significance of EIF4ENIF1 splicing variant in the diagnosis and management of patients with POI or EM. This newly discovered variant was identified for the first time in POI family with incomplete penetrance, providing novel molecular genetic evidence for EIF4ENIF1 variants in POI or EM pathogenesis.

## Introduction

Female reproductive aging is a continuum characterized by the depletion of ovarian follicles. While Premature Ovarian Insufficiency (POI) is the most severe manifestation, defined by hypergonadotropic hypogonadism and amenorrhea before the age of 40, presenting as amenorrhea or oligomenorrhea lasting for at least 4 months, accompanied by elevated follicle-stimulating hormone (FSH) levels (>25 IU/L) and reduced estradiol levels (Webber et al., 2016; Panay et al., 2020). POI affects approximately 3.1%–4.3% of the female population worldwide (Nash and Davies, 2024). It is important to distinguish POI from related conditions. Early Menopause (EM) is defined as menopause occurring between 40 and 45 years of age (Hamoda and Sharma, 2024). Diminished ovarian reserve (DOR) is characterized by reduced antral follicle count (AFC) (e.g., AFC < 5–7), low anti-Müllerian hormone (AMH) levels (e.g., AMH < 1.2 ng/mL) in the presence of normal FSH levels and regular menstrual cycles (Grisendi et al., 2019; Penzias et al., 2020). These conditions may represent a continuum of ovarian dysfunction with varying degrees of severity (Lafraoui et al., 2024).

The etiology of POI/EM/DOR is complex, involving autoimmune, metabolic, iatrogenic, infectious, environmental, psychological, and genetic factors (M and R, 2019). Among these contributors, genetic defects are considered pivotal determinants in POI pathogenesis, accounting for up to 30% of idiopathic POI cases and exhibiting substantial locus heterogeneity (Rossetti et al., 2017; Alvarez-Mora et al., 2020). In recent years, the widespread application of Whole Exome Sequencing (WES) has facilitated the identification of multiple candidate disease-causing genes associated with POI. To date, over 100 pathogenic genes have been confirmed, including *FOXL2*, *GDF9*, *TP63*, *NOBOX* and others (Federici et al., 2024). Despite the evident genetic predisposition of POI, its high degree of genetic heterogeneity means that individual genes account for only a small subset of affected cases (Xue et al., 2018). Consequently, the underlying etiology remains unclear in a considerable number of POI patients.

Eukaryotic Translation Initiation Factor 4E Nuclear Import Factor 1 (EIF4ENIF1) is an RNA-binding protein (RBP) that contains a binding site for the mRNA 5′cap-binding protein eIF4E (Sonenberg and Hinnebusch, 2009). As a rate-limiting factor for cap-dependent translation initiation, EIF4ENIF1 can negatively regulate translation by binding to eIF4E (Kamenska et al., 2014a). Notably, EIF4ENIF1 is essential for nuclear membrane rupture in mammalian oocytes and the resumption of meiosis (Pfender et al., 2015). Previous studies have indicated that EIF4ENIF1 is the pathogenic gene of clinical POI, and its heterozygous knockout mice exhibit POI and infertility symptoms (Ding et al., 2023; Moriwaki et al., 2025).

Pre-mRNA splicing is a critical process for removing non-coding introns and is essential for human gene expression (Lee and Rio, 2015). Accurate splicing relies on the precise recognition of donor and acceptor splice sites at the 5′ and 3′ ends of intron-exon junctions. The canonical 5′ splice site contains the dinucleotide “GU”, which is recognized by U1 snRNP (Zhang et al., 2021); while the 3′ splice site comprises the “AG” dinucleotide, which is recognized by U2 snRNP (van der Feltz and Hoskins, 2019). Generally, intronic variants that disrupt the GT-AG dinucleotide consensus sequence impair pre-mRNA splicing (Padgett, 2012). To date, the vast majority of pathogenic splicing variants have been identified at the intronic GT-AG dinucleotide sites (Krawczak et al., 1992).

In this research, we recruited a family affected by POI/EM and employed WES to investigate the genetic cause within this lineage. A rare heterozygous EIF4ENIF1 (NM_019843) variant was identified: c.1769–2A>G. Our findings revealed that the proband’s mother, maternal grandmother, and sister also carried this variant. While a proband may present with signs suggestive of ovarian dysfunction, family members may exhibit a heterogeneous phenotype—ranging from frank POI to EM or asymptomatic DOR, highlighting the importance of evaluating relatives for the broader spectrum of ovarian aging, including DOR and EM, rather than focusing solely on classical POI criteria.

## Materials and methods

### Patients

Patients were diagnosed with POI, EM, DOR from The Second Xiangya Hospital of Central South University. All procedures involving human participants were conducted in accordance with the ethical standards of the hospital’s ethics committee, as well as the Declaration of Helsinki (1964) and its subsequent amendments. Written informed consent was obtained from each participant in this study.

### WES analysis

Genomic DNA was extracted from peripheral blood of the recruited participants using the QIAamp DNA Mini Kit (Qiagen, Valencia, CA, USA) and the amplicons were gel-purified and sequenced by Salus Pro platform (ShenZhen SalusBiomed Ltd). Variant filtering was performed through a two-step process. First, rare and novel variants with minor allele frequency (MAF) < 1% were selected, and MAFs were analyzed using data from the Exome Aggregation Consortium (ExAC, http://exac.broadinstitute.org/), 1000 Genomes Project (http://browser.1000genomes.org/index.html), the Genome Aggregation Database (http://gnomad.broadinstitute.org/), and the ESP6500 database (http://evs.gs.washington.edu/EVS/). Second, only frameshift, nonsense, missense, and splice site variants were retained.

### Sanger sequencing verification

WES results were validated through Sanger sequencing. For the EIF4ENIF1 (c.1769–2A>G) variant, PCR amplification and Sanger sequencing were performed using forward (5′-GAT​GGC​GTT​CAG​CAA​GAA​ATG-3′) and reverse (5′-GGC​AAG​CAA​GAG​ATT​GAG​TTT​G-3′) primers. DNA products were assessed by electrophoresis on an ABI 3730 XL DNA sequencer (Applied Biosystems, Bedford, MA).

### Splicing site analysis

Homologous nucleic acid and amino acid sequence alignments and visualizations were performed using Jalview 2.11.2.5 (https://www.jalview.org/download/). Premature stop codons within mutant transcripts and amino acid sequences were identified using the Open Reading Frame Finder (ORFfinder) from NCBI (https://www.ncbi.nlm.nih.gov/orffinder/).

### RNA isolation, cDNA synthesis, cDNA amplification and sequencing

Fresh blood samples from the proband, her affected sisters and healthy controls were collected and stored in PAXgene Blood RNA Tubes (BD Bioscience). Total RNA was isolated using the PAXgene Blood RNA Kit and assessed using the Qubit 2100. Genomic DNA was removed from the total RNA, and the RNA was subsequently reverse transcribed into cDNA using the HiScript III First Chain cDNA Synthesis Kit (gDNA wiper, Vazyme). Forward (5′-AGT​GCC​GTA​ACC​AAC​AAT-3′) and reverse (5′-GGG​TAC​AGA​GCT​GGA​TGA-3′) primers were employed for PCR amplification and Sanger sequencing.

### Protein structure prediction

AlphaFold2 (https://colab.research.google.com/github/sokrypton/ColabFold/blob/main/AlphaFold2. ipynb) was employed to predict the 3D structure of the mutated EIF4ENIF1 protein. The Protein Data Bank (PDB) file for the wild-type EIF4ENIF1 protein was retrieved from the AlphaFold Structure Database (https://alphafold.ebi.ac.uk/). Secondary structure predictions were generated using PSIPRED, which utilizes feedforward neural networks in conjunction with PSI-BLAST outputs to construct position-specific scoring matrices. The 3D structures of both mutant and wild-type EIF4ENIF1 proteins were visualized and compared using PyMOL 2.2.3.

## Results

### Clinical profile of the POI/EM family

The proband (III-2), a 36-year-old female, presented with a 4-year history of infertility. Detailed menstrual history revealed regular menstrual cycles (cycle length 30–34 days, menstrual duration 3–5 days). Hormonal assessment showed FSH 10.66 IU/L, LH 8.06 IU/L, AMH 2.2 ng/mL, and estradiol 34.57 pg/mL. At age 36, her AMH level of 2.2 ng/mL (within the normal range for her age group) (Table 1). These observations do not provide evidence to support a diagnosis of DOR or POI in the proposita. Furthermore, her mother (II-2) experienced oligomenorrhea and amenorrhea at the ages of 37 and 39, respectively, a condition that could align with POI, although other potential causes of secondary amenorrhea have not been definitively excluded. Additionally, her maternal grandmother (I-2) experienced oligomenorrhea at 38 and amenorrhea at 41, a pattern that more closely resembles EM. Her younger sister (III-3) is 26 years old, with normal menstruation.

**TABLE 1:** Clinical profiles of the proband.

Parameters	Value
Ultrasound imaging: Endometrial thickness	D3 Em 3.0 mm
The longest diameter of the ovary (right/left) mm	R 30 × 21 mm L
Follicles	R 4-5f L display unclear L
Follicle-stimulating hormone, IU/L	10.66
Luteinizing hormone, IU/L	8.06
Estrogen, pg/mL	34.57
AMH, ng/mL	2.20
PRL, ng/mL	14.66
Progesterone, ng/mL	0.38
Testosterone, ng/mL	27.9

### WES analysis and sanger sequencing of the POI/EM family

**FIGURE 1:**
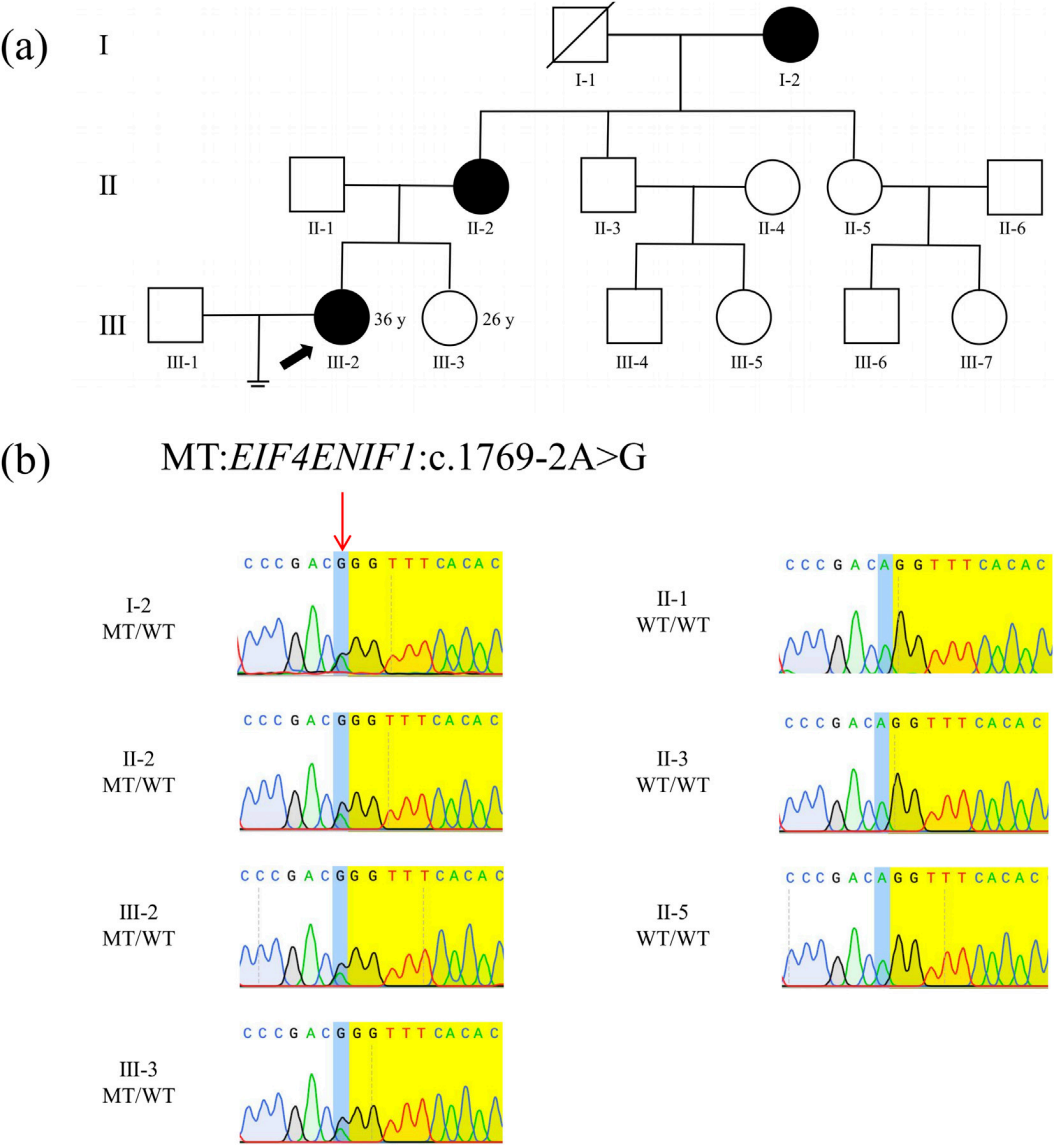
The pedigree of the proband and Sanger sequencing of patients carrying the EIF4ENIF1 c.1769–2A>G variant. **(a)** The pedigree of the family. Squares denote male family members, circles female family members, and solid symbols affected members; slashes denote deceased family members, equal signs infertility, and the double line infertility. The arrow indicates the index patient (family member III-2). **(b)** Normal sequencing profiles and profiles for the heterozygous variant (G/A) at codon1769-2 in EIF4ENIF1. Four family members carried a heterozygous variant, and three were wild-type.

WES was performed on the proband, which identified a novel variant, c.1769–2A>G, in the POI-associated gene EIF4ENIF1. To further validate this finding and investigate the distribution of this single nucleotide variant (SNV) within the family, Sanger sequencing was conducted on additional family members (Figure 1). Among the individuals tested, the proband’s mother (II-2), maternal grandmother (I-2), and younger sister (III-3) were found to carry the same heterozygous variant in EIF4ENIF1. The proband and her younger sister (III-2 and III-3) are at risk to potentially develop POI or EM and may also be at risk of DOR, reflecting the continuum between these conditions. Genetic testing provides critical guidance for their genetic counseling and clinical management: early surveillance with regular hormonal monitoring and AMH measurements, and AFC assessments via transvaginal ultrasound, counseling on elective fertility preservation (e.g., oocyte cryopreservation) prior to ovarian function decline, and individualized reproductive planning to reduce the risk of involuntary childlessness.

### Pathogenicity analysis of the EIF4ENIF1 variants

**FIGURE 2:**
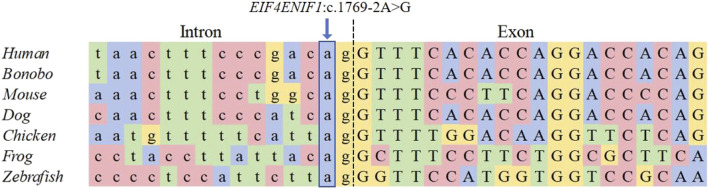
Sequence alignment analysis of EIF4ENIF1 gene in multiple species. The panel shows the alignment analysis of the c.1769–2A site within the EIF4ENIF1 gene between humans, bonobo, mouse, dog, chicken, frog and zebrafish.

The intronic GT-AG dinucleotide site plays a crucial role in pre-mRNA splicing. Through sequence alignment analysis, we observed that the c.1769–2A site within the EIF4ENIF1 gene exhibits high sequence conservation across multiple species, ranging from humans to zebrafish (Figure 2). In these species, the 3′splice sites of EIF4ENIF1 strictly follow the canonical “AG” dinucleotide rule. Therefore, we hypothesize that the c.1769–2A>G variant may disrupt the normal splicing of the EIF4ENIF1 gene. In silico predictions using different algorithms (dbscSNV, variant Taster, CADD, and fathmm-MKL) also suggest that the c.1769–2A may be a pathogenic variant (Table 2).

**TABLE 2:** In silico prediction of c.1769–2A>G in EIF4ENIF1.

Functional prediction	Result
dbscSNV_ADA_SCORE	1
dbscSNV_RF_SCORE	0.938
variantTaster_score	1
variantTaster_pred	D
CADD_raw	4.846
CADD_phred	33
Fathmm-MKL_coding_score	0.968
Fathmm-MKL_coding_pred	D
GERP++_NR	6.16
GERP++_RS	6.16

### Identification of exon 11 skipping caused by the splicing variant

**FIGURE 3:**
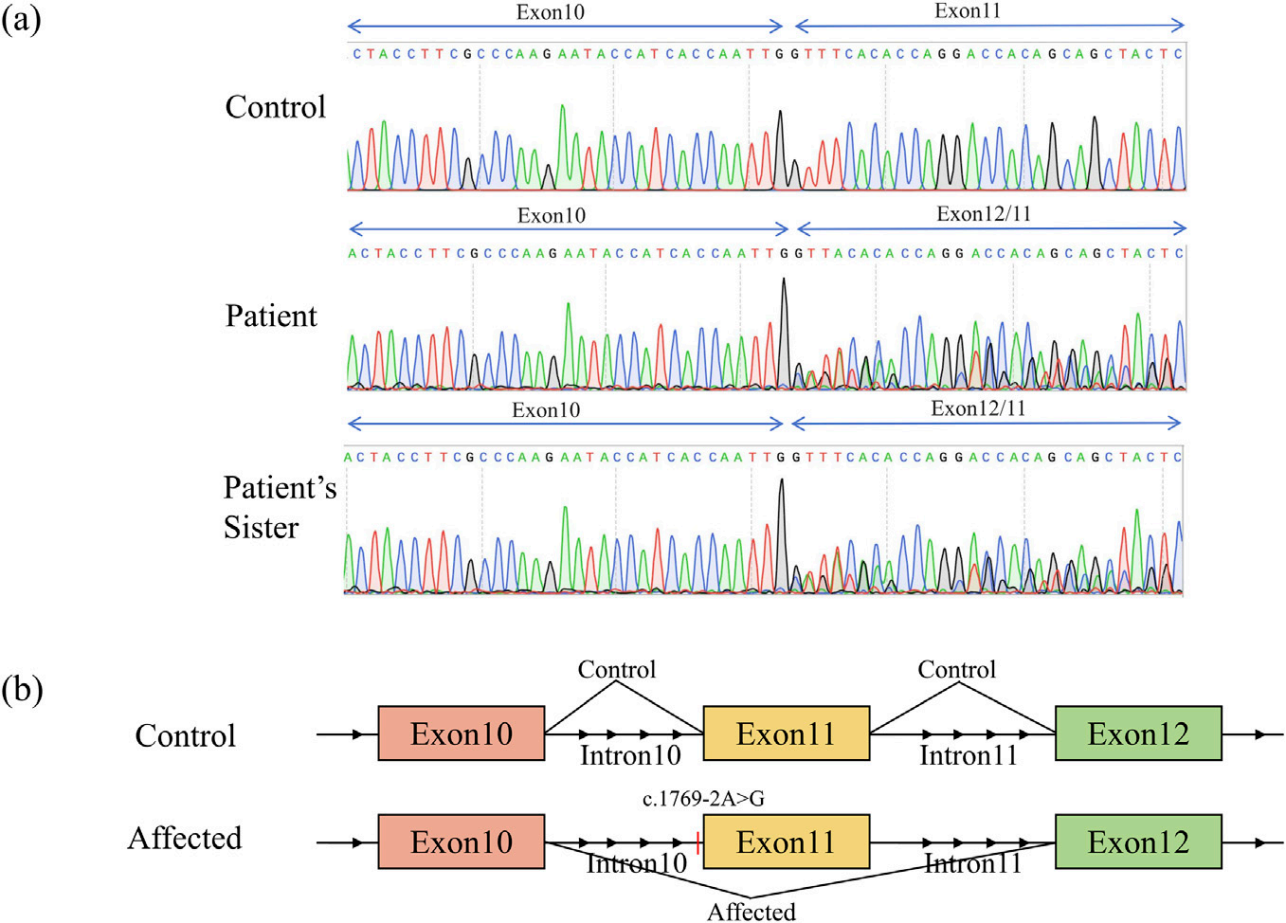
Identification of the Splicing Defect Caused by the c.1769–2A>G Variant. **(a)** Sanger sequencing results for control, patient, and patient’s sister, showing sequence variations at splice sites; **(b)** Schematic diagram of splicing mechanism shows abnormal splicing in affected individuals with exon 11 skipped and the c.1769–2A>G variant annotated, compared to normal splicing process that exons 10–11–12 connected sequentially.

To investigate the impact of the c.1769–2A>G variant on EIF4ENIF1 gene splicing, peripheral blood RNA from the patient and her sister was reverse transcribed into cDNA, followed by amplification of specific regions. Using RT-PCR combined with Sanger sequencing, we conducted a detailed analysis of EIF4ENIF1 mRNA splicing patterns in both individuals. Through sequence alignment analysis, we matched the amplified sequences from the variant carrier with those from the healthy control group (Figure 3a). Sanger sequencing revealed that amplification of the EIF4ENIF1 gene in the c.1769–2A>G variant carrier produced abnormal transcripts, exhibited an exon 11 skipping event, compared to the healthy control group with normal splicing process that exons 10–11–12 connected sequentially (c.1768_1769delGTTTCACACCAGGACCACAGCAGCTACTCGGAGATCCATTCCAAGGCATGCGCAAACCCATGAGCCCCATCACAGCCCAG) (Figure 3b).

### Impact of the variant on protein

**FIGURE 4:**
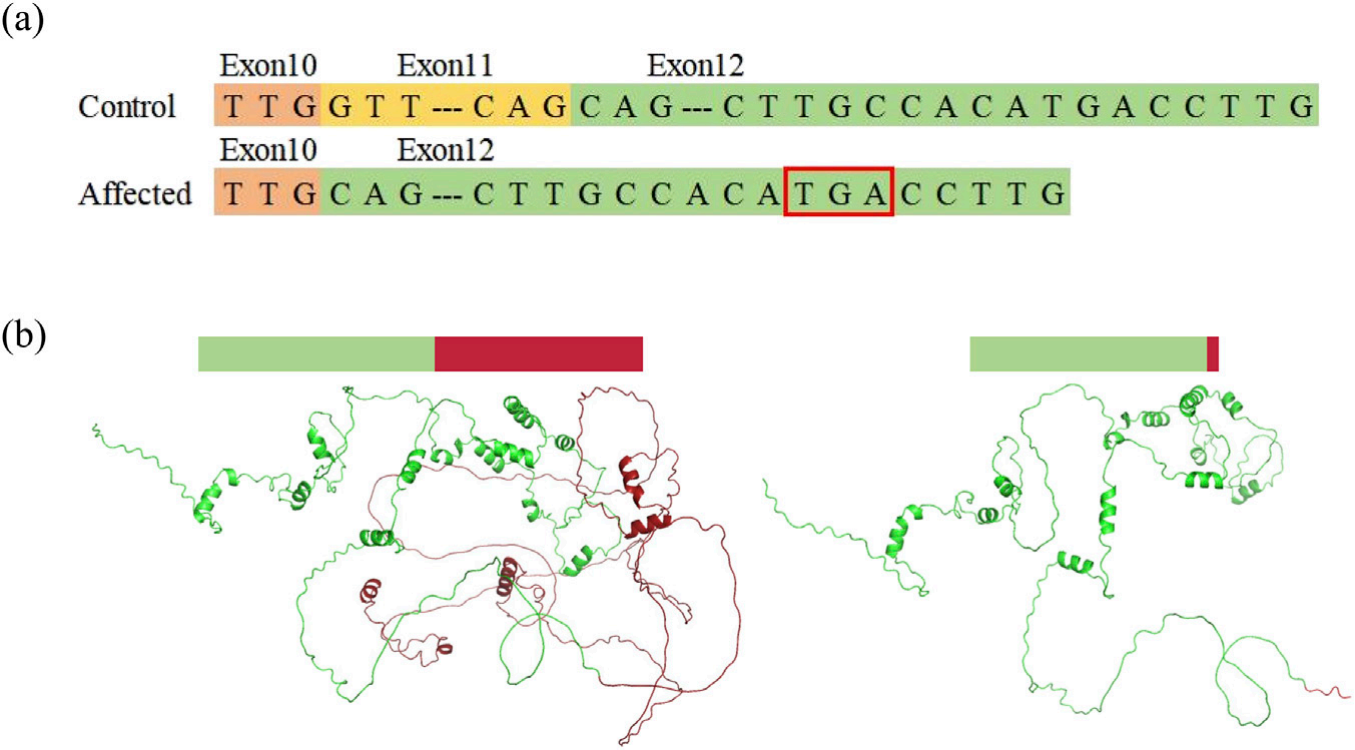
Gene Sequence Analysis and Protein Structure Prediction. **(a)** Gene sequence analysis: control displays normal gene sequence comprising exons 10, 11, and 12 with intact splicing between exons; affected individual exhibits exon 11 skipping, directly connecting exon 10 to exon 12; the red-boxed “TGA” denotes a stop codon; this skipping induces a reading frame shift, resulting in premature termination. **(b)** Protein structure prediction: left panel shows the normal protein with complete structure and green regions representing normally folded protein domains; right panel shows the mutant truncated protein with markedly incomplete structure lacking crucial domains.

We further assessed the impact of exon 11 skipping in the EIF4ENIF1 gene on the encoded protein. Exon 11 spans 80 base pairs (bp), and its deletion may result in premature occurrence of the stop codon, thereby causing premature truncation of the protein (p.Ile589fs*18). Analysis of the mutated cDNA sequence using the ORF finder tool revealed an earlier position of the terminator codon (Figure 4a) within the mutated sequence, suggesting that this variant may result in truncation of the EIF4ENIF1 protein. By comparing the protein sequences of wild-type and truncated EIF4ENIF1, we identified a deletion of 378 amino acid residues in the mutant subtype. This alteration significantly impacts the structure and function of the EIF4ENIF1 protein. Furthermore, we employed AlphaFold 2 modelling to predict the structure of the EIF4ENIF1 protein. Results revealed substantial structural alterations in the truncated protein (Figure 4b). These structural changes may compromise the functional integrity of the EIF4ENIF1 protein, thereby disrupting its involvement in cellular biological processes.

### EIF4ENIF1 variants review

To date, variants in EIF4ENIF1 associated with POI are relatively rare in reported human cases, with only six variants described, encompassing both truncating and missense variants (Table 3). The novel variant identified in this study provide additional insights into the mutational spectrum and potential clinical implications of EIF4ENIF1 dysfunction.

**TABLE 3:** Six variants linking EIF4ENIF1 to POI.

Protein variant	Nucleotide change	References
S429Term	1286C>G	Kasippillai et al. (2013)
Q842P	2525A>C	Zhao et al. (2019)
K669R	2006A>G	França et al. (2020)
R4del	9_11delGAG	Shang et al. (2022)
G954A	2861G>C	Shang et al. (2022)
R208H	623G>A	Din et al. (2024)

## Discussion

In this study, we report a novel heterozygous intronic variant c.1769–2A>G in the EIF4ENIF1 gene associated with POI/DOR/EM. Through family genetic analysis, we determined that the patient inherited this variant from her mother, and the patient’s maternal grandmother and sister similarly carry this variant. Variant analysis using bioinformatics tools indicated that the c.1769–2A>G variant is highly likely to be a pathogenic allele. Furthermore, this variant directly impacts the canonical GT-AG splice receptor site, resulting in 11 skipping and consequently causing truncation of the EIF4ENIF1 protein.

EIF4ENIF1, as a RBP, is primarily localized in the ribonucleoprotein complex P-body in the cytoplasm (Kamenska et al., 2014b). In developing oocytes, EIF4ENIF1 exhibits high expression levels (Villaescusa et al., 2006). This protein participates in the formation of the Mitochondrial-Associated Ribonucleoprotein Domain (MARDO) within oocytes and P-body like germ granules in early-stage female germ (Flemr et al., 2010; Cheng et al., 2022). It has been reported that EIF4ENIF1 functions as an EIF4E-binding protein with translational inhibitory activity. Ding et al. constructed POI-associated EIF4ENIF1 mutants in somatic cell lines, demonstrating that these variants impaired the translational suppression function of EIF4ENIF1 to varying degrees (Ding et al., 2024).

EIF4ENIF1 is essential for the recovery of nuclear envelope breakdown (NEBD) and meiosis in oocytes. Researchers observed that downregulation of EIF4ENIF1 expression in developing follicles leads to meiotic arrest at the germinal vesicle (GV) stage of oocytes (Pfender et al., 2015). Ding et al. further generated a heterozygous EIF4ENIF1 knockout mouse model, revealing the impact of haploinsufficiency on mouse fertility (Ding et al., 2023). The study found that insufficient EIF4ENIF1 protein function impaired mouse fertility, while homozygous knockout mice exhibited embryonic lethality. From 9 months of age, *EIF4ENIF1*-deficient mice displayed significantly reduced follicle numbers and a mild decrease in litter size. At both transcriptional and translational levels, *EIF4ENIF1*-deficient oocytes exhibited maturation defects and dysregulated gene expression, alongside disrupted mitochondrial dynamics. Furthermore, the distribution of the MARDO was abnormal within these oocytes.

These findings suggest that haploinsufficiency may underlie the effects of heterozygous EIF4ENIF1 variants (Kasippillai et al., 2013; Zhao et al., 2019). EIF4ENIF1 inhibits protein translation by binding to eIF4E; reduced EIF4ENIF1 levels lead to partial suppression of eIF4E inhibition. This may result in increased protein translation and dysregulated gene expression (Ding et al., 2024), subsequently impairing follicular development and causing POI.

RNA splicing, as a critical step in the regulation of gene expression in eukaryotes, involves the removal of introns and the joining of exons within pre-mRNA (Chen and Manley, 2009). Within this process, the precise recognition of splice donor sites and splice acceptor sites is essential for the normal functioning of the splicing mechanism. These sites exhibit a high degree of evolutionary conservation, reflecting their importance in maintaining accuracy gene expression (Black, 2003).

Variants at splice sites frequently disrupt normal pre-mRNA splicing patterns, leading to abnormal exon splicing such as skipping and the aberrant activation of cryptic splice sites. These splicing abnormalities form the molecular basis for numerous genetic diseases (Kurmangaliyev and Gelfand, 2008). According to variant classification guidelines issued by the American College of Medical Genetics and Genomics (ACMG) and the Association for Molecular Pathology (AMP), disruption of the highly conserved GT-AG splicing motif (±1 or 2 nucleotides from the splice site) is considered strong evidence for pathogenicity (Richards et al., 2015; Chen and Manley, 2009). Literature estimates suggest that approximately 10% of pathogenic variants involve splicing abnormalities, with variants at these splicing sites associated with a significant proportion of inherited disorders (Krawczak et al., 2007). In this study, we identified the c.1769–2A>G variant in the EIF4ENIF1 gene. This variant damaged the 3′end AG splice acceptor site within intron 10, thereby disrupting mRNA splicing. This led to exon 11 skipping, impairing EIF4ENIF1 protein function and resulting in the patient’s symptoms.

DOR, POI, and EM may be regarded as different clinical stages along a continuum of premature ovarian functional decline. DOR mainly reflects a reduction in the quantity of recruitable follicles or a reduced ovarian response, while menstrual cycles and basal hormone levels may still be relatively preserved (Pastore et al., 2018; Younis et al., 2020). With further depletion of the follicular pool and endocrine decompensation, affected women may develop POI, characterized by menstrual disturbance before the age of 40 years together with elevated FSH levels. If ovarian dysfunction progresses to permanent cessation of menstruation before the age of 45 years, the phenotype may present as EM. Thus, these conditions are not completely separate entities, but rather represent a clinical spectrum ranging from diminished ovarian reserve to ovarian insufficiency and eventually early ovarian failure (Tucker et al., 2016).

In this family, the coexistence of infertility with borderline ovarian reserve markers, suspected POI, early menopause, and asymptomatic carriers supports the concept of a phenotypic continuum with incomplete and age-dependent penetrance (Alvarez-Mora et al., 2020). The proband presented with infertility but did not currently meet the diagnostic criteria for overt POI. This genotype–phenotype discrepancy may be explained by incomplete penetrance, whereby not all carriers of the POI-related variant develop a clinically recognizable POI phenotype (Shekari et al., 2023). In addition, because POI and early menopause are age-related manifestations of ovarian functional decline, the absence of typical POI features in the proband at present may reflect age-dependent penetrance or an early stage of phenotypic expression. Consequently, the proband may be at increased risk of developing POI before 40 years of age or early menopause later in life. A recommendation for genetic counseling and regular follow-up, including menstrual assessment, reproductive hormone testing and ultrasonographic evaluation of ovarian reserve is necessity for her (Panay et al., 2024).

It should be explicitly acknowledged that the proband’s mother experienced amenorrhea at 39 years of age and the maternal grandmother ceased menstruating at 41 years of age, yet neither underwent hormonal evaluation at the time of ovarian function decline. Given the long interval since their menopause, retrospective measurement of FSH, LH, estradiol, or AMH would not faithfully reflect their endocrine status at the onset of amenorrhea and therefore carries no diagnostic utility for POI or EM. This lack of biochemical data precludes definitive classification of the observed amenorrhea as POI, DOR, or EM and constitutes a substantial limitation when inferring familial aggregation patterns. The interpretation of the pedigree should be accordingly conservative.

Within the study cohort, one young carrier (namely, the proband’s sister, III-3) aged 26 years has not yet exhibited clinical manifestations POI, EM or DOR. In light of this, we recommend that she undergoes regular medical monitoring (serial AMH, FSH, and AFC assessments) to enable timely detection of any potential pathological changes, or considers pursuing pregnancy at an appropriate juncture to avoid missing the fertility window. The variable expressivity among carriers in this pedigree exemplifies the characteristic incomplete penetrance of monogenic POI disorders: up to 50% of heterozygous EIF4ENIF1 pathogenic variant carriers remain asymptomatic into their 30s or 40s, with phenotypic severity influenced by modifier genes, epigenetic regulation, and environmental factors (Shekari et al., 2023). This underscores the necessity of lifelong reproductive counseling and longitudinal follow-up for at-risk relatives even in the absence of overt clinical manifestations.

In summary, this study identifies a novel pathogenic variant in EIF4ENIF1 that disrupts the splice receptor site at intron 10 and induces exon 11 skipping. The deletion of exon 11 results in premature termination codons, leading to truncation of the EIF4ENIF1 protein and subsequent dysfunction. To date, this represents the first intron-affecting variant in the EIF4ENIF1 gene identified in patients with POI/DOR/EM. It expands the spectrum of EIF4ENIF1 gene variants, providing novel molecular genetic evidence for understanding the role of EIF4ENIF1 gene variants in POI and EM pathogenesis. The genetic architecture of ovarian insufficiency and early menopause involves both rare pathogenic variants and common polygenic contributions, highlighting the importance of comprehensive genetic analysis in affected families (Alvarez-Mora et al., 2020). This finding holds significant clinical implications for genetic counselling and diagnosis of POI-related diseases.

## Data Availability

The datasets presented in this study can be found in online repositories. The names of the repository/repositories and accession number(s) can be found in the article/supplementary material.
